# On the Use of a Signal Quality Index Applying at Tracking Stage Level to Assist the RAIM System of a GNSS Receiver

**DOI:** 10.3390/s16071029

**Published:** 2016-07-02

**Authors:** Mattia Berardo, Letizia Lo Presti

**Affiliations:** Politecnico di Torino—Corso Duca degli Abruzzi 24, Torino 10129, Italy; letizia.lopresti@polito.it

**Keywords:** Global Navigation Satellite System (GNSS), signal processing, Receiver Autonomous Integrity Monitoring (RAIM), integrity

## Abstract

In this work, a novel signal processing method is proposed to assist the Receiver Autonomous Integrity Monitoring (RAIM) module used in a receiver of Global Navigation Satellite Systems (GNSS) to improve the integrity of the estimated position. The proposed technique represents an evolution of the Multipath Distance Detector (MPDD), thanks to the introduction of a Signal Quality Index (SQI), which is both a metric able to evaluate the goodness of the signal, and a parameter used to improve the performance of the RAIM modules. Simulation results show the effectiveness of the proposed method.

## 1. Introduction

Global Navigation Satellite Systems (GNSS), like Global Positioning System (GPS) or Galileo, are getting more and more pervasive in many fields, including aviation, railway, maritime, and road. In order to satisfy the integrity requirements, becoming crucial for many applications, these systems do not only need to be precise and accurate, but they have to guarantee solutions with a certain level of reliability. In the last years, GNSS integrity increased its relevance in all the transportation sectors, especially for Safety of Life (SoL) services and liability critical applications, such as, for instance, road tolling applications, pay-as-you drive insurance, and other commercial services. However, the integrity algorithms for land applications are relatively new and not completely assessed, due to the fact that the number of new services continues to rapidly grow.

Nowadays, the technology of the integrity systems in aviation is mature and many algorithms have been developed for this specific application. However, the absence of obstacles, the distance between the airborne and possible interference sources from the Earth, a reduced occurrence of multipath (MP) and the good visibility of the satellites make the techniques for aviation not directly applicable to other applications. In fact in sectors, such as road, railway, rural, and maritime, the environmental conditions around the receiver are in general worse than in aviation, and this implies that the integrity algorithms for aviation have to be modified. An example of integrity concepts applied to a urban environment can be found in [1].

A well assessed integrity technique is the so-called Receiver Autonomous Integrity Monitoring (RAIM) [2], which can be implemented by adopting different algorithms, such as range comparison [3], Pseudorange (PR) residual or Residual Based (RB) [4], and parity method [5]. They provide snapshot schemes and yield identical results under the assumption of equal alarm rate, as shown in [6], where a detailed comparison of these methods is given. Another well known RAIM scheme is the Solution Separation that works on the position domain [7]. A basic assumption of all these schemes is that only one fault at a time is possible. However, these schemes can be extended to the case of multiple faults, as described in the recent work [8].

The RAIM algorithms use PR and position data to implement the Fault Detection and Exclusion (FDE) module, which leads to the exclusion of faulty satellites in the position computation. However the identification of faulty satellites can be done also at the tracking stage of the receiver, by adopting adequate signal processing techniques, as shown, for example, in the recent paper [9]. Today, these methods cannot be implemented in a current commercial GNSS receiver, but a new generation of receivers can be devised with new blocks working in the correlation domain, and able to assist the FDE module of RAIM.

The work presented in this paper analyses the feasibility of this concept of assisted-RAIM. In particular it is focused on the effects of environmental impairments, as MP, and how to assist RAIM to protect the receiver from this type of errors. MP occurs in the presence of objects, close to the receiver, which can reflect the signals coming from the satellites. The working principle of a GNSS receiver is based on the measurements of the delay between the time instant when a signal leaves the satellite and the time instant when the signal is received. Therefore, if reflected signals are received together with the useful signals, an error on the estimation of the delay of the GNSS signals may occur. The Delay Lock Loop (DLL) is the block devoted to the delay estimation. However a DLL error not always impairs the PR measurements. This fact has to be taken into account in the design of assistance methods based on correlation points estimated by a DLL. More details on this aspect are given in Section 3.

In the literature, many MP mitigation techniques are proposed (e.g., [10,11], just to cite a couple of examples). However, when the goal is to satisfy some integrity requirements, the general approach is to identify the presence of errors in the delay estimation without mitigating its effect, but rather excluding the satellite affected by impairments from the solution. We propose here to adopt a technique of MP detection, which derives from the MP Distance Detector (MPDD) described in [12], and based on the identification of a Linear Adaptive Filter (LAF) able to model the MP effects, as shown in [13,14]. The new version of MPDD described in this paper introduces a Signal Quality Index (SQI), which is both a metric able to evaluate the goodness of the signal, and a parameter used to improve the performance of the FDE module of RAIM. Note that methods of Signal Quality Monitoring (SQM) are already proposed in literature in the framework of integrity systems. For example in [15,16] SQM algorithms are proposed to detect anomalous signal distortions by using extra correlators, which are not used to maintain the receiver locked to the incoming signal. The difference here is that the signal quality is used to create an index (SQI), which becomes an integral part of the assisted RAIM.

The paper is organised as follows: in Section 2 a brief introduction of the considered GNSS receiver architecture and its working principles is given. In Section 3 the methods of PR calculation are reviewed and the MP impact on DLL outputs and PR measurements is analysed. In Section 4 an overview of the LAF theory used in [12] is given. Section 5 is devoted to the description of the MP detector. Section 6 introduces the concept of SQI. In Section 7 some RAIM and FDE concepts are given and the RB RAIM algorithm is briefly explained. In Section 8 a possible interaction between SQI and RAIM is described and simulation results are given. Section 9 concludes the paper.

## 2. Receiver Architecture

It is known that a GNSS signal in space (SIS) is transmitted by using a Code Division Multiple Access (CDMA) format and the received signal of the GPS L1 C/A code can be written as a combination of the SISs of all the satellites in view, each one expressed by

(1) $$y_{m}\left(nT_{s}\right)=\sqrt{2P_{m}}C_{m}\left(nT_{s}-\tau_{m}\right)d_{m}\left(nT_{s}-\tau_{m}\right)\cos\left(2\pi \left(f_{IF}+f_{d,m}\right)nTs+\varphi_{m}\right)+w\left(nTs\right)$$

where *m* is the index of a specific satellite, $P_{m}$ is the received power, $C_{m}\left(\cdot \right)$ is the Pseudo Random Noise (PRN), $d_{m}\left(\cdot \right)$ is the navigation data, $f_{IF}$ is the intermediate frequency of the front-end, $f_{d}$ is Doppler frequency, $\varphi_{m}$ is a phase term, $\tau_{m}$ is the code delay and $T_{s}$ is the sampling interval. The second term of the sum is a noise component w(nTs), that is a discrete-time random process obtained by sampling the noise at the front-end output. Since the input noise may be assumed to be White Gaussian Noise (WGN) with power spectral density $S\left(f\right)=N_{0}/2$ at the front-end output, w(nTs) becomes a Gaussian discrete-time random process with zero mean and variance $\sigma^{2}=N_{0}B_{IF}$, where $B_{IF}$ is the front-end bandwidth. Therefore Equation (1) represents a so called Additive White Gaussian noise (AWGN) channel. Thanks to the code orthogonality, the receiver can discriminate different signals coming from different satellites. Therefore the analysis of the receiver can be done considering only a single signal $y_{m}\left(nT_{s}\right)$.

In our experiments we used the receiver architecture described in [17], and in particular we implemented a distortion-detector working at the tracking stage. The detector structure is strictly related to the working principle of the DLL and to the mechanism of PR measurements which are described in Section 2 and Section 3.1.

### DLL

The main purposes of the DLL is to maintain a local code $C_{loc,m}\left[n\right]$ of the receiver locked to the incoming PRN code $C_{m}\left[n\right]$. To do this, DLL estimates the relative delay between two codes by using a correlation operation. However, the typical implementation of the DLL is not based on the estimation of a delay, rather, on the estimation of a code frequency $f_{c}$. To maintain locked $C_{loc,m}\left[n\right]$ and $C_{m}\left[n\right]$, every integration time $T_{int}$ (i.e., 1 ms), the output of the DLL’s discriminator is used to correct $f_{c}$ and this mechanism yields to have a variable number of samples every $T_{int}$. The presence of filters in the loop introduces a delay in the receiver chain or better, a transient with a duration dependent on the filter bandwidth: narrow band means less noise but long transient and vice versa. To align the incoming PRN code with a locally generated code, a DLL must include integrators, a code loop discriminator, and code loop filters. In our scheme the signal Equation (1) is first demodulated by multiplying it by two local carrier waves at frequency $f_{IF}$, one for each branch of the receiver (I and Q). The resulting signal is multiplied by three local codes (called early, prompt, late), generally shifted by −1/2, 0 and +1/2 chip time, and the outputs are integrated and dumped to implement the correlator.

The model chosen for the discrimination function is a *non-coherent* scheme, independent of the phase of the local carrier, and with the expression

$$\frac{\left(I_{e}^{2}+Q_{e}^{2}\right)-\left(I_{l}^{2}+Q_{l}^{2}\right)}{\left(I_{e}^{2}+Q_{e}^{2}\right)+\left(I_{l}^{2}+Q_{l}^{2}\right)}$$

called Normalised Early minus Late power, where $I_{e}=AR_{x}\left(\tau -d/2\right)\cos\left(\phi_{e}\right)$, $Q_{e}=-AR_{x}\left(\tau -d/2\right)\sin\left(\phi_{e}\right)$, $I_{l}=AR_{x}\left(\tau +d/2\right)\cos\left(\phi_{e}\right)$, $Q_{l}=-AR_{x}\left(\tau +d/2\right)\sin\left(\phi_{e}\right)$, and $R_{x}\left(\cdot \right)$ is the correlation function between the local and incoming code, *A* is an amplitude factor, *τ* is the code delay of the signal, $\phi_{e}$ is the estimated carrier phase error and *d* is the correlator spacing between early and late.

We are interested in investigating what happens on the DLL parameters, when a disturbance, e.g., an MP, affects the received signal.

## 3. PR Calculation in a GNSS Receiver

From the theory, we know that a typical GNSS receiver computes the user’s position from the estimated PR $\rho_{i}$ (distance between the *i*-th satellite and the receiver). Considering that both satellites and receiver’s clocks are affected by independent errors, we call *system time* the reference time frame where satellites and the receiver’s clocks are referred. Before introducing the equation, we define some notations [17]:

- $T_{t}$ is the system time at which the signal left the satellite
- $T_{RX}$ is the system time at which the signal reached the user receiver
- δt is the offset of the satellite clock from system time (written in the navigation message)
- $t_{RX}$ is the offset of the receiver clock from system time
- $T_{t}+\delta t$ is the satellite clock reading at the time that the signal left the satellite
- $T_{RX}+t_{RX}$ is the user receiver clock reading at the time the signal reached the user receiver
- *c* is the speed of light

A generic *ρ* for a single satellite is given by taking into account all the clock errors

(2) $$\begin{array}{cc} \rho & =c\;\left(T_{RX}+t_{RX}\right)-c\;\left(T_{t}+\delta t\right)\\ & =c\;\left(T_{RX}-T_{t}\right)+c\;\left(t_{RX}-\delta t\right)\\ & =r+c\;(t_{RX}-\delta t) \end{array}$$

where *r* is the geometric range and the second term $c\;(t_{RX}-\delta t)$ is the residual distance due to the not perfect synchronisation between satellite and receiver clocks. To calculate this, the time of flight of the signals is measured and the clock corrections will be made after data demodulation and Position, Velocity and Time (PVT) computation.

### 3.1. How the GNSS Receiver Implements the PR Computation

In order to process tracked signals independently, GNSS receivers assign each one a dedicated channel. As said, to obtain *ρ* it is necessary to know the transmitted time $T_{t}$ for each satellite and to correct all the misalignments of the receiver and the satellite clock with respect to the system time. Two possible implementations of this measurement are Common transmission time and Common reception time [18,19]. The former one is based on the satellites’ transmission time. Obviously the channels are not synchronised each other at the receiver side, so on each channel the same bit of a subframe (and relative time of arrival of it) have to be identified. The receiver selects a reference channel by using the first arriving bit, and calculates the relative delay $\Delta_{i}$ with respect to the bit of *i*-th satellite, $\Delta_{i}=T_{RX,i}-T_{RX,1}$. This allows to write the range $r_{i}$ of the *i*-th satellite as $r_{i}=\rho_{1}+c\;\delta t_{i}+c\;\Delta b+c\;\Delta_{i}$, where $\rho_{1}$ is the PR of the reference channel or in other words, the satellite closest to the user, Δb is the unknown bias for the not perfect synchronisation between clock on board the *i*-th satellite and the clock of the receiver. Finally, $\delta t_{i}$ is the correction of the offset of the *i*-th satellite clock from system time.

In the second implementation (Common reception time) the receiver calculates $\Delta_{i}=T_{u}-T_{RX,i}$, where, in this case, $\Delta_{i}$ is the time interval between the instant of reception of the subframe for the *i*-th channel $T_{RX,i}$ and $T_{u}$, that is the common receiving time when the receiver decides to measure the PR over all channels. All the PRs are derived with respect to the reference channel, which is the one with the minimum travel time. Once $\Delta_{i}$ are computed for all the channels, $r_{i}=\rho_{1}+c\;\Delta b+c\;\left(\Delta_{1}-\Delta_{i}\right)+\delta t_{i}$.

To measure the time interval $\Delta_{i}$, the receiver continuously counts the samples processed per each channel and maintains a monotone counter in 20 ms increments derived from receiver’s reference oscillator [17]. The mechanism of counting partially protects the PR measurements from correlation distortions. In next section this is proved by simulation since a theoretical explanation would require a deep analysis of both DLL and counting operations.

The Common reception time is the method usually employed in commercial GNSS receivers.

### 3.2. Simulation Experiments

In this section we investigate which is the effect of the correlation distortions on the PR measurements. In fact it is known that the PRs are measured in scheduled time epochs with a typical rate of 1 Hz (or few Hz), while a DLL computes correlations with a much greater rate (e.g., 50 Hz). Therefore, we expect that a distortion in the correlation may have a different impact if it occurs during the local code update performed by the DLL, or at the epochs of PR measurements. To analyse these different effects we have performed some simulation tests. The simulation scenarios were created by using the signal generator described in [20]. The analyzed datasets represent a static position with four satellites in view all affected by one-reflected ray of MP. The received signal is GPS L1 C/A, it lasts 60 s with different values of Carrier-to-Noise ratio ($C/N_{0}$), but constant for the whole duration.

The dimension of the time windows of the MP events are different, so as to test the impact of the duration on the accuracy reached by the receiver on the PVT stage. The MP model used in the simulation is

$$\begin{array}{cc} s\left(t\right)=A_{0}D\left(t-\tau_{0}\right)C\left(t-\tau_{0}\right)\cos(2\pi (f_{IF} & +f_{d}\left(t\right))t+\phi \left(t\right))\\ & +\sum_{i=1}^{N}A_{i}D\left(t-\tau_{i}\right)C\left(t-\tau_{i}\right)\cos\left(2\pi \left(f_{IF}+f_{d_{i}}\left(t\right)\right)t+\phi_{i}\left(t\right)\right) \end{array}$$

where $A_{0}$ is the signal amplitude of the Line-of-Sight (LOS) and $A_{i}$ is the amplitude of the reflected rays. The Doppler frequency, $f_{d}\left(t\right)$, is variable in time but, to simplify the simulations, all the Doppler frequencies of the MPs are equal to the Doppler of the LOS ($f_{d_{i}}\left(t\right)=f_{d}\left(t\right)$). The values of the initial phase and delay of the reflected ray are randomly chosen. All the satellites in view are affected by a simulated single ray MP during the time windows indicated in Table 1. The Multipath-to-Signal Ratio (MSR) is the parameter used to assign the MP amplitude. It is defined as the ratio between the power of the MP signal and the power of signal itself. MSR is constant and equal to −6 dB (about half amplitude of the signal) for all the time windows.

### 3.3. Preliminary Results

Since the purpose of the simulation experiments is to compare the effect of MP on the measured PR, two versions of the simulated signal are created:

(a) the signal $s_{c}\left(t\right)$, which represents the clean scenario, containing only noise;
(b) the signal $s_{m}\left(t\right)$ obtained by adding MP to $s_{c}\left(t\right)$ in the time windows given in Table 1.

**Figure 1:**
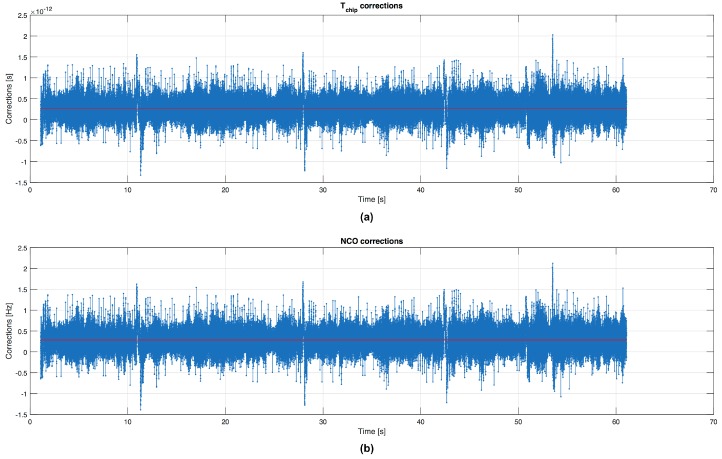
$\Delta T_{chip}\left[n\right]$ in time (**a**) and corrections of code frequency $\Delta f_{c}\left[n\right]$ for PRN 31 (**b**).

**Figure 2:**
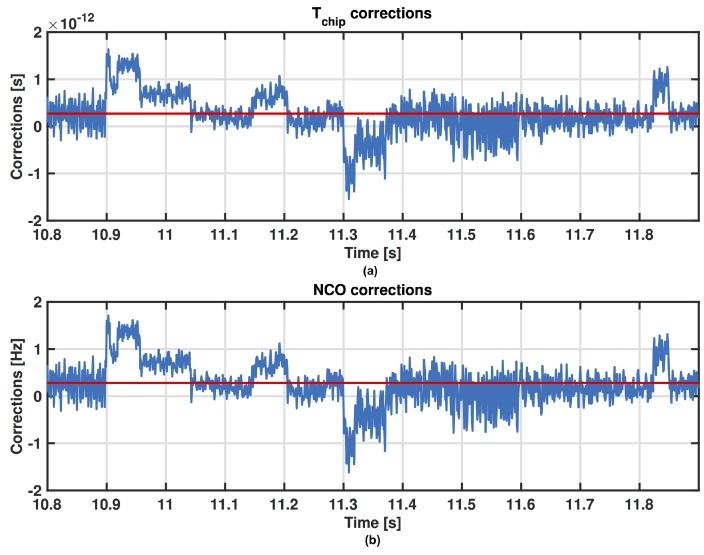
Zoom of the $\Delta T_{chip}\left[n\right]$ (**a**) and $\Delta f_{c}\left[n\right]$ (**b**) for PRN 31 in the first time window of Table 1.

**Figure 3:**
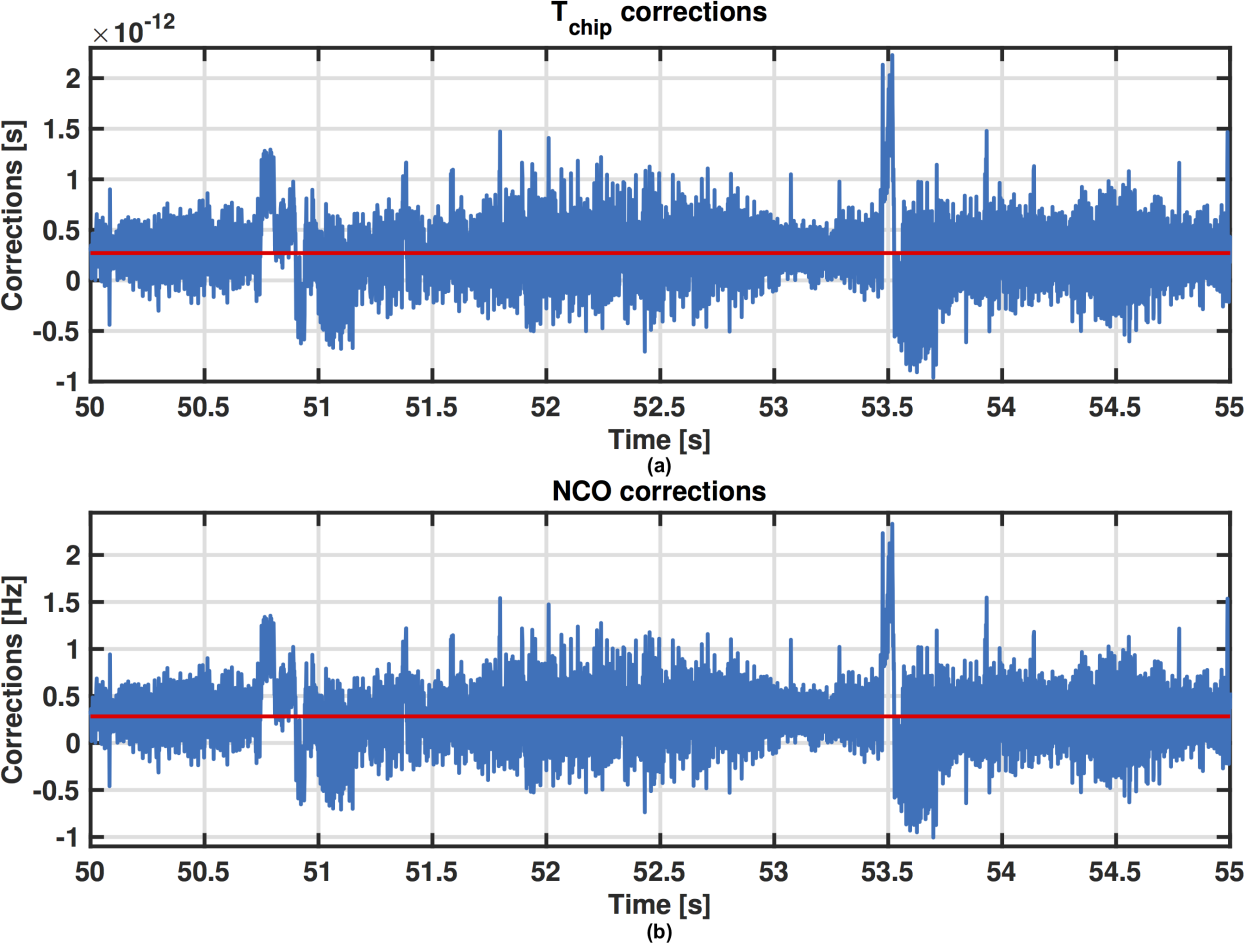
Zoom of the $\Delta T_{chip}\left[n\right]$ (**a**) and $\Delta f_{c}\left[n\right]$ (**b**) for PRN 31 in the last time window of Table 1.

Figure 1 shows the time evolution of the code frequency corrections $\Delta f_{c}\left[n\right]=f_{c}\left[n+1\right]-f_{n}$ where $f_{n}$ is the nominal value of the code frequency (1.023 MHz in GPS L1 C/A), and the relative value in time calculated as $\Delta T_{chip}\left[n\right]=T_{chip}-1/f_{c}\left[n+1\right]$ in the DLL. The red line is the average value for the corrections. Let’s start by analysing this figure in the time windows specified in Table 1. What we want to observe is the behaviour of the PR computation just before and during a MP occurrence. The MP effect is clearly observed in the first time window shown in Figure 2, where a change of the code frequency appears during the disturbed time window. Then after a transient, the estimate of the code frequency returns to be stable. Figure 3 shows similar results in time windows affected by MPs with different durations.

To make some comparisons with simulated data, we calculated PR $\rho_{clean}$ in a scenario with only noise and PR $\rho_{MP}$ in a scenario with noise and MP in the time windows given in Table 1. Then, we computed the difference $\rho_{clean}-\rho_{MP}$ in order to see the impact of tracking errors at PR level. These operations are repeated for different values of the output rate $T_{\rho }$ of the PR measurements. The persistent distortions could introduce errors on the estimate of $f_{c}$ and consequently in PR computation based on samples counting (as we explained in Section 3.1).

**Figure 4:**
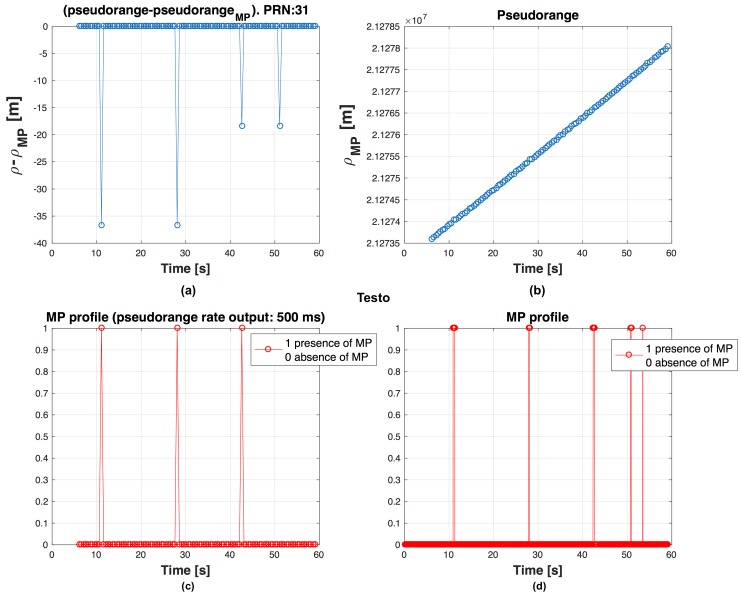
Behaviour of PR for PRN 31, with output every $\Delta T_{\rho }=500$ ms. PR error $\left(\rho_{clean}-\rho_{MP}\right)$ (**a**); $\rho_{MP}$ trend in time (**b**); MP presence during a PR computation (**c**) and MP profile (**d**) are shown.

**Figure 5:**
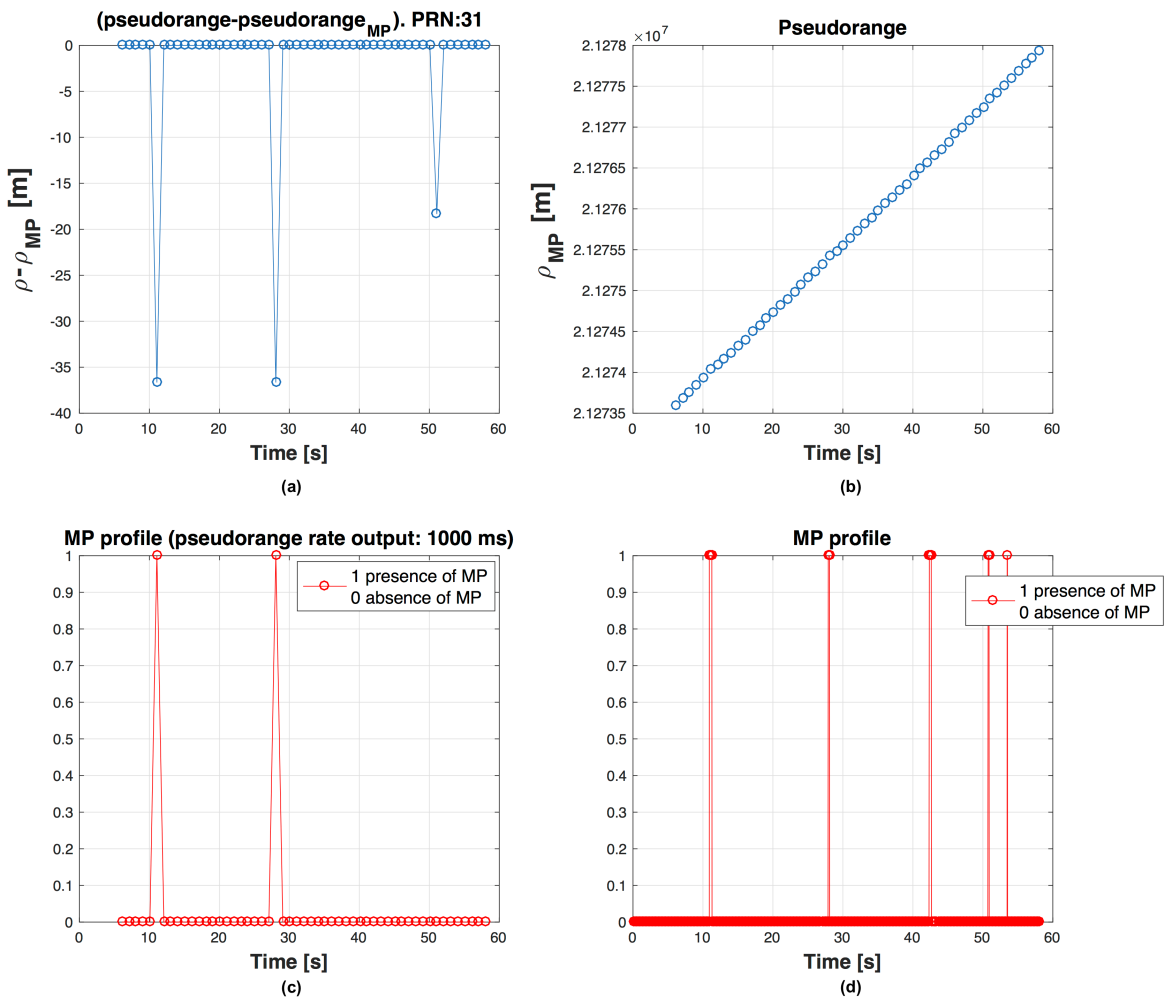
Behaviour of PR for PRN 31, with output every $\Delta T_{\rho }=1000$ ms. PR error $\left(\rho_{clean}-\rho_{MP}\right)$ (**a**); $\rho_{MP}$ trend in time (**b**); MP presence during a PR computation (**c**) and MP profile (**d**) are shown.

**Figure 6:**
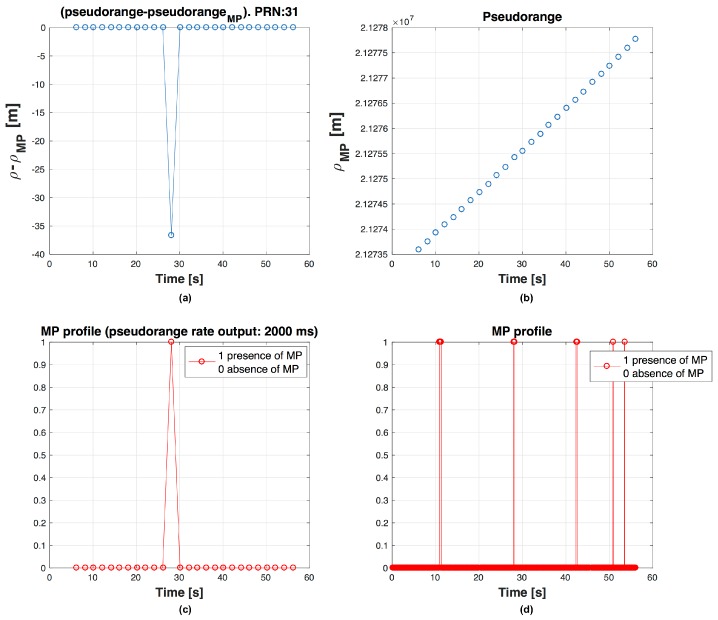
Behaviour of PR for PRN 31, with output every $\Delta T_{\rho }=2000$ ms. PR error $\left(\rho_{clean}-\rho_{MP}\right)$ (**a**); $\rho_{MP}$ trend in time (**b**); MP presence during a PR computation (**c**) and MP profile (**d**) are shown.

We observe that, if we use short $T_{\rho }$, affected by MPs limited in time, the probability that next PR calculated is wrong is higher than if we use longer $T_{\rho }$. It is possible to observe this effect in Figure 4, Figure 5 and Figure 6, where on the top left graph there is the difference $\rho_{clean}-\rho_{MP}$, on the top right the evolution of the $\rho_{MP}$ in time, at bottom left the graph shows if the PR is measured in presence of MP and at bottom right the MP profile. For example there are respectively 3, 2 and 1 PRs measured during MP event in Figure 4, Figure 5 and Figure 6. Errors in code frequency $f_{c}$ estimation may alternate the PR computation. These errors can be recovered by the DLL if the duration of the distortion is limited in time and, once the disturbance is finished, there is enough time before next PR measurement for the DLL to recover the error.

**Figure 7:**
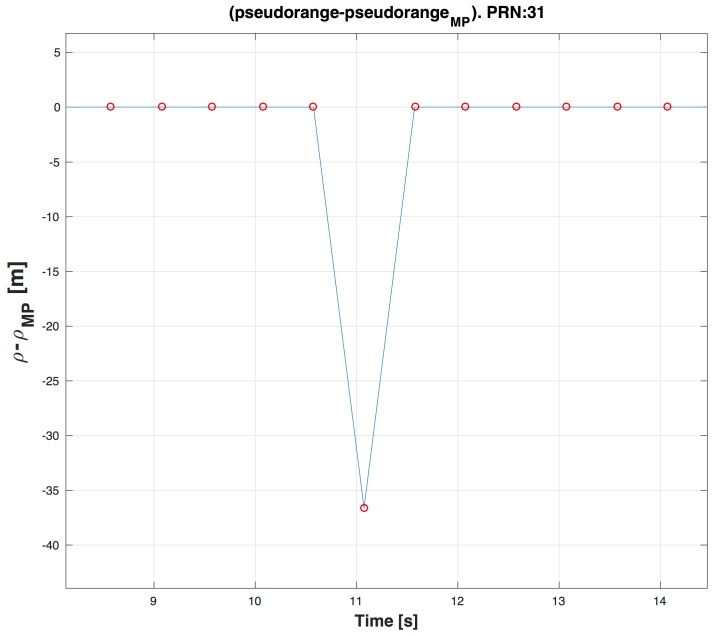
Zoom around 10 s of $\rho_{clean}-\rho_{MP}$ in the case $\Delta T_{\rho }=500$ ms in blue and MP profile in red.

In Figure 7 a zoom of the Figure 4 is depicted, where the circle points indicate that a MP is present in a time instant of PR computation. As expected the quantity $\rho_{clean}-\rho_{MP}$ is different from zero where circle points are present.

## 4. Overview of Linear Adaptive Filter Techniques

The MP detector presented in this paper derives from the technique described in [12], based on the Linear Adaptive Filter (LAF) theory. A brief description of the LAF methods is given here, to motivate the new version of the MP detector described in this paper.

A complete overview of *adaptive algorithms* is available in [21], where adaptive techniques are presented for channel equalisation. In particular LAF methods are described in [22,23]. In the LAF theory, a generic measured signal is modeled as $d\left[n\right]=y\left[n\right]+n_{0}\left[n\right]$, where $n_{0}\left[n\right]$ is a noise sequence and y[n] is

$$y\left[n\right]=\sum_{k=0}^{M-1}w_{k}^{*}u\left[n-k\right]$$

where u[n] is a reference signal, and $w_{k}^{*}$ are the coefficients of a Finite Impulse Response (FIR) filter, which provides an approximated version y[n] of the input signal d[n]. The length of the filter *M* represents the number of delayed components that decompose the incoming signal. The LAF coefficients $w_{k}^{*}$ are estimated by minimising the cost function

(3) $$\epsilon =\sum_{n=i_{1}}^{i_{2}}{|e\left[n\right]|}^{2}$$

that is the error energy, where e[n]=y[n]−d[n] is the *residual error*, and $i_{1}$ and $i_{2}$ are the limits of the sum. The values assigned to the limits depend on which kind of *data windowing* is chosen. In [12] we use the so-called covariance method, where $i_{1}=M$ and $i_{2}=N$, and no assumption is made on the data outside the interval [1,N]. With this approach the minimisation problem can be solved in matrix form, after introducing the quantities

(4) $$U^{H}=\left[\begin{array}{cccc} u(M) & u(M+1) & \cdots & u(N)\\ u(M-1) & u(M) & \cdots & u(N-1)\\ \vdots & \vdots & \ddots & \vdots \\ u(1) & u(2) & \cdots & u(N-M+1) \end{array}\right]$$

$$\Phi =U^{H}U$$

and

$$z=U^{H}D$$

where $D={\left[d\left[M\right],d\left[M+1\right],\cdots ,d\left[N\right]\right]}^{T}$. It is possible to prove that the vector of estimated coefficients $\hat{w}={\left[w_{0},\cdots ,w_{M-1}\right]}^{T}$ can be written as

(5) $$\hat{w}=\Phi^{-1}z$$

In this way, the problem of estimating $\hat{w}$ turns in an inversion matrix problem, by assuming **Φ** is nonsingular, therefore invertible.

Another approach to window the data is to define the matrix U as

(6) $$U^{H}=\left[\begin{array}{ccccccc} u(1) & u(2) & \cdots & u(N) & u(N) & \cdots & u(N)\\ u(1) & u(1) & \cdots & u(N-1) & u(N) & \cdots & u(N)\\ \vdots & \vdots & \ddots & \vdots \\ u(1) & u(1) & \cdots & u(N-M+1) & u(N-M) & \cdots & u(N) \end{array}\right]$$

where some a priori assumptions are made, simply replicating the value u(1) before $i_{1}=1$ and replicating the value u(N) after $i_{2}=N$, as in Equation (6). In this paper we describe a method based on this approach, whose advantages are explained in Section 5. The output provided by the LAF method is then used to define a test statistic for distortions detection. Therefore the ‘adaptation’ of the coefficients at each new measurement allows us to continuously update the value of the test statistic.

## 5. MPDD Algorithm

The MP detector described in this paper works in parallel with the tracking stage, because it uses the raw data after demodulation and the correction terms calculated by the DLL. The detector does not influence the normal operations of the receiver, but raises an alert when MP or, more in general, distortions affect the signal.

In principle the structure of the multicorrelator used in the detector can be different from the one used in the DLL. However MPDD will have an impact on the computational complexity of blocks working at the tracking layer, since it requires a number of additional correlation points with respect to a single DLL. The applicability of this scheme to specific GNSS receivers will be considered in future work, since this paper is only focused on a feasibility study. In particular we are planning to work on the possibility to distribute the evaluation of the correlation points in different epochs, so as to make the method feasible also for consumer grade GNSS receivers (of course reduced performance is expected).

The detector is composed of four elements:

1. *Multicorrelator*, which computes *N_points_* of the correlation between the received GNSS code, and a local code
2. *Moving average filter*, which smoothes the noise effect on the points generated by the multicorrelator
3. *Linear adaptive filter*, which estimates the MP contribution in the measured correlation
4. *Decision metric*, which decides if the measured correlation is either distorted or clean.

The number of points *N_points_* computed by the multicorrelator impacts on the computational load and on the resolution achieved in the estimation of the delay created by a MP. Therefore the choice of *N_point_* will be a result of a trade-off between complexity and resolution. Another critical point is the effect of noise, which degrades the quality of the measured correlation. To mitigate the effect of noise a moving average filter is used to smooth the input of the LAF block. Two possible implementations of this filter are continuous moving average or block moving average. The first one is a moving average filter with overlap between slices of the signal, while the second one evaluates the average of independent slices, without overlapping. To reduce the computational load we decided to adopt the block moving average [12]. The LAF module decomposes the input correlation in a weighted sum of triangles (ideal correlation of GPS L1 C/A) and estimates the vector $\hat{w}$ of the filter taps. The last step analyses $\hat{w}$ and declares if some distortion is present. The results presented in [12], based on the *covariance method* exploit the information provided only in the right side of the peak of the correlation. Therefore, the technique handles positive delays with respect to the LOS and limits the number of used taps being U in Equation (4) dependent on *M*. The LAF-based method presented here uses the data windowing in Equation (6), containing additional points to avoid loosing data points.

### Least Squares and Constrained Least Squares

In [12], the Least Squares (LS) problem was simply the minimisation of the error *ϵ* in Equation (3). To reduce false alarms caused by noise, an updated version has been developed corresponding to the problem:

(7) $$\begin{array}{ccc} \text{min} & & \epsilon \\ \text{subject}\;\text{to} & & w_{i}>0,\;i=1,\ldots ,M. \end{array}$$

This is a Constrained Least Squares (CLS) minimisation of Equation (3) with the constraint that the coefficients of the filter are all positive. This means that only positive ideal correlations are used to model the measured correlation. We can adopt this model even if we know that components in counter-phase introduce negative correlations. The negative data will be erroneously modelled, since the method is constrained to assign positive weights to each correlation replica. If the replica is negative the algorithm will construct multiple weights in an effort to minimise the minimum Mean Square Error (MSE). This does not allow the correct identification of the negative replica, but the multiple weights indicate that a replica exists, and this is sufficient for detecting the presence of anomalies.

The decision metric is the same of the original algorithm, so we compute the square distances between $\hat{w}$ and a dictionary of sample vectors *V*. By observing these distances we are able to decide if the correlation function presents distortions. The dictionary *V* takes into account different scenarios, including LOS condition with no extra signals ($w_{LOS}$) and multipath with multiple rays ($w_{k}$).

$$E_{LOS}={∥\hat{w}-w_{LOS}∥}^{2}$$

and

$$E_{k}={∥\hat{w}-w_{k}∥}^{2}\;k=1,2,\ldots N_{v}-1$$

where $N_{v}$ is the number of vectors in the dictionary *V*. The anomalies are present if

$$E_{k}<E_{LOS}\;k=1,2,\ldots N_{v}-1$$

or

$$\min_{k=1,2,\ldots N_{v}-1}\left(E_{k},E_{LOS}\right)\neq E_{LOS}$$

The advantage of CLS is the reduction of the possible entries of the dictionary in the decision phase.

The CLS minimization adopted here is based on the Non-Negative Least Squares, method described in [24]. In the presence of noise the traditional LS estimates the input noise as a weighted sum of delayed replicas of the input signal, which is a suitable model for the MP channel but does not fit well to the WGN channel. As a consequence, the system adds a high number of positive and negative $w_{i}$ coefficients in order to model the WGN contribution. The result is a highly noisy solution. A constraint is then put here on the possible values of the $w_{i}$ coefficients. In particular, they can be only positive. As already said, this constraint appears not suitable to estimate a MP channel where LOS and MP components have different phase sign. In such cases, in order to properly estimate the signal components, negative $w_{i}$ coefficients would be needed. However, it has to be noted that the goal of the algorithm is not to properly estimate the signal as a weighted sum of components, but to detect if anomalies are present in the signal, discriminating between the only-LOS case and all the other unwanted cases. If a counter-phase component is present, then the CLS estimates non-null $w_{i}$ coefficients with shorter delay with respect to the LOS. Such an anomalous behaviour detects the presence of counter-phase MP.

In case of only LOS presence without noise, the LS and CLS solutions are equivalent. In general, the CLS algorithm will give less non-null coefficients than LS.

## 6. SQI

The output provided by the detector is a hard detection (Yes/No) about the presence of correlation anomalies. However it could be interesting to introduce a sort of soft decision to better assist the RAIM.

The idea has been to continuously monitor the quality of the received signal during the computation of navigation solution. To achieve this goal we introduced an index $\text{SQI}(t_{n})$ that describes the quality of the navigation solution, where $t_{n}$ are the time instants of the PVT computation.

**Figure 8:**
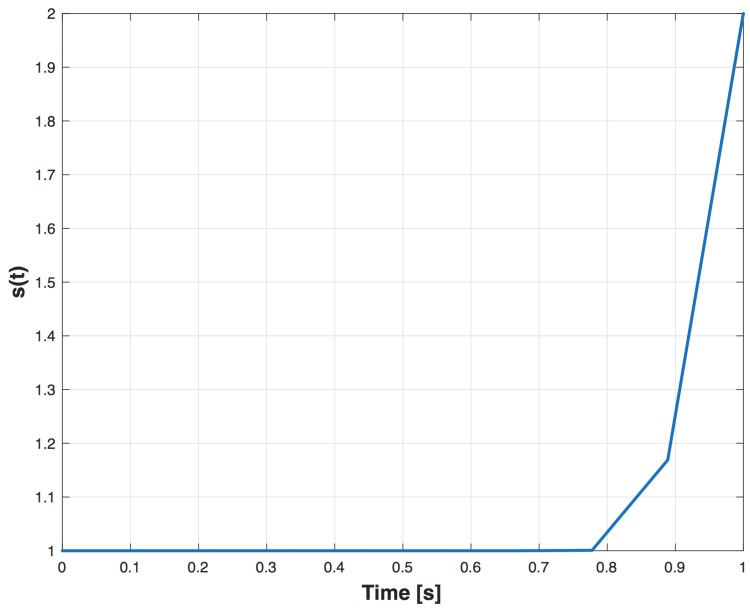
Trend of s(t) within two instants of the PVT computation.

**Figure 9:**
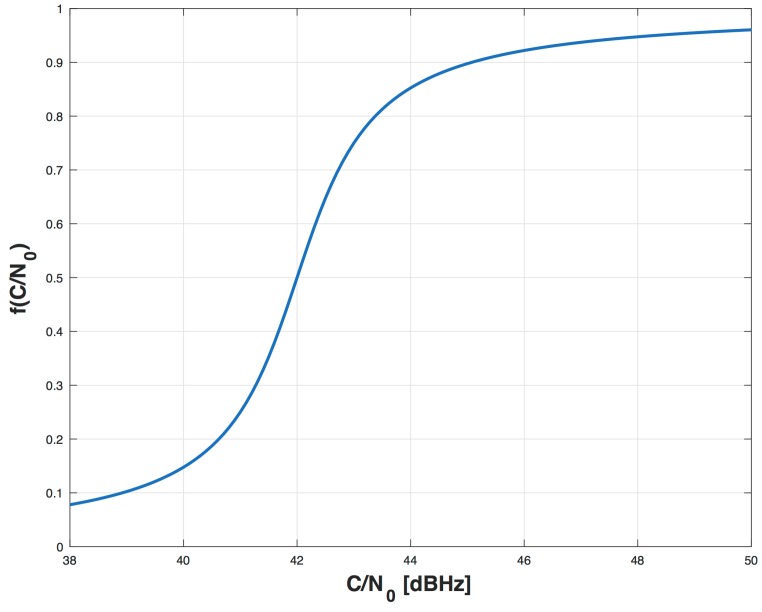
Example of trend of *f* function with a=42 dBHz.

This quality index takes into consideration the MPDD output and other information data such as the $C/N_{0}$ and the distance between the weight vector $\hat{w}$ and the theoretical LOS vector $w_{LOS}$. It is defined as

$$\text{SQI}\left(t_{n}\right)=\frac{1}{\lambda }\sum_{k=1}^{N}m\left(t_{n-1}+kT_{a}\right)s\left(t_{n-1}+kT_{a}\right)d\left(t_{n-1}+kT_{a}\right)f\left[\frac{C}{N_{0}}\left(t_{n-1}+kT_{a}\right)\right]$$

where:

- $T_{a}$ is the duration of the moving average time window.
- *N* is the number of the moving average time windows between two time instants ($t_{n}$ and $t_{n-1}$). For example, between two PVT computations with a rate of 1 Hz and a moving average of $T_{a}$ = 100 ms, N=10.
- m(·) represents the MPDD output,
  $$m\left(kT_{a}\right)=\left\{\begin{array}{cc} -1, & \text{if}\;E_{min}=\min E_{p}=E_{LOS},\;\;p=1,2,\ldots N_{V}\\ 1, & \text{if}\;E_{min}=\min E_{p}\neq E_{LOS},\;\;p=1,2,\ldots N_{V} \end{array}\right.$$
  meaning that −1 indicates a correlation function with at most noise presence, instead 1 the presence of distortions.
- The function s(·) takes into account the temporal distance between the occurrence of the distorting event and the epoch when the PVT is computed. In fact the PVT module of any GNSS receiver uses the estimated PRs at some given epochs, and, as explained in Section 3.3, correlation distortion between two epochs could have an impact on the PR estimation. The meaning of the function s(·) is to give a different importance to the MPDD output measurements depending on how far they are from the next PR computation. s(·) is a Gaussian function normalised and shifted by 1 in order to have a range of the values from 1 to 2. The shape of s(·) is shown in Figure 8.
- The function d(·) measures the ratio $E_{min}/E_{LOS}$, and it is defined as
  $$d\left(kT_{a}\right)=\left\{\begin{array}{cc} E_{min}/E_{LOS}, & \text{if}\;E_{min}\neq E_{LOS}\\ 1, & \text{if}\;E_{min}=E_{LOS} \end{array}\right.$$
  d(·) is a ratio between distances, so it gives a relative measure of how far is $\hat{w}$ with respect to the LOS.
- f(·) takes into account the $C/N_{0}$ value, and is defined as:
  $$f\left[\frac{C}{N_{0}}\left(kT_{a}\right)\right]=\frac{1+\frac{2}{\pi }\arctan\left[\frac{C}{N_{0}}\left(kT_{a}\right)-a\right]}{2}$$
  The $C/N_{0}$ gives a measure of the reliability of the output of the MPDD. As a matter of fact, if $C/N_{0}$ is high, it means that the decision about the presence of distortions is more reliable than with a $C/N_{0}$ closer to *a*. The variable *a* is the minimum operative $C/N_{0}$ considered by the algorithm. We chose an arcotangent function as shown in Figure 9 to take into account a saturation mechanism in case of very high or very low $C/N_{0}$.
  It is important to choose a $C/N_{0}$ estimator that suits our problem. In [25] a comparison between five well-known methods to estimate the $C/N_{0}$ is made. We chose to use an estimator with low computational complexity, the *Signal-to-Noise Variance* (SNV), that is based on the first absolute moment and the second moment of the signal samples [25].
- *λ* is a normalization factor to obtain $\text{SQI}\left(t_{n}\right)\in \left(0,1\right]$. The parameter *λ* is set as the sum of the maximum values that can be obtained at each discrete time *k* for the functions m(·), s(·), d(·) and f(·).

## 7. RAIM

Traditional RAIM techniques provide integrity information, by making use of the available measurements [2]. The basic assumption for RAIM is the presence of only one fault at the same time. This is a strong assumption, but the process may be iterated so including *de facto* the case of multiple faults. In literature three main RAIM algorithms are proposed: range comparison, least-squares residuals and parity method. All the methods are able to determine the presence of a satellite failure by using the redundancy of the measurements of the over-determined system of linearised equations

Δρ=HΔx+ϵ

where Δx is a vector containing the incremental deviations of the user position from the linearisation point, H is the geometry matrix, where the generic row *j* is the unit vector pointing from the linearisation point to the considered *j*-th satellite, and *ϵ* is the vector of PR errors. To detect position errors, RAIM requires at least five satellites in view, while the exclusion of the faulty measurements requires at least six satellites. The snapshot approach means that the system takes decision about the presence of failure by using only current considered observations.

In this paper, we propose a method of assisted RAIM, based on the idea to detect satellite failures at tracking stage level, since errors like MP are much more visible in the correlation rather than at the PR level.

### 7.1. Global and Local Test

The structure of a RAIM algorithm comes from the statistical detection theory [2]. Two hypothesis tests are posed: Global and Local Test. The first one tests if a failure exists and the second one identifies which is the failed satellite, under the assumption to have at most one failure at a time. In the residual method, the first step is to compute the residual between predicted and measured PRs [26], $\hat{r}=H\Delta \hat{x}-\Delta \rho$, given by

$$\hat{r}=H{\left(H^{T}\Sigma^{-1}H\right)}^{-1}H^{T}\Sigma^{-1}\Delta \rho -\Delta \rho =-R\Delta \rho$$

**Figure 10:**
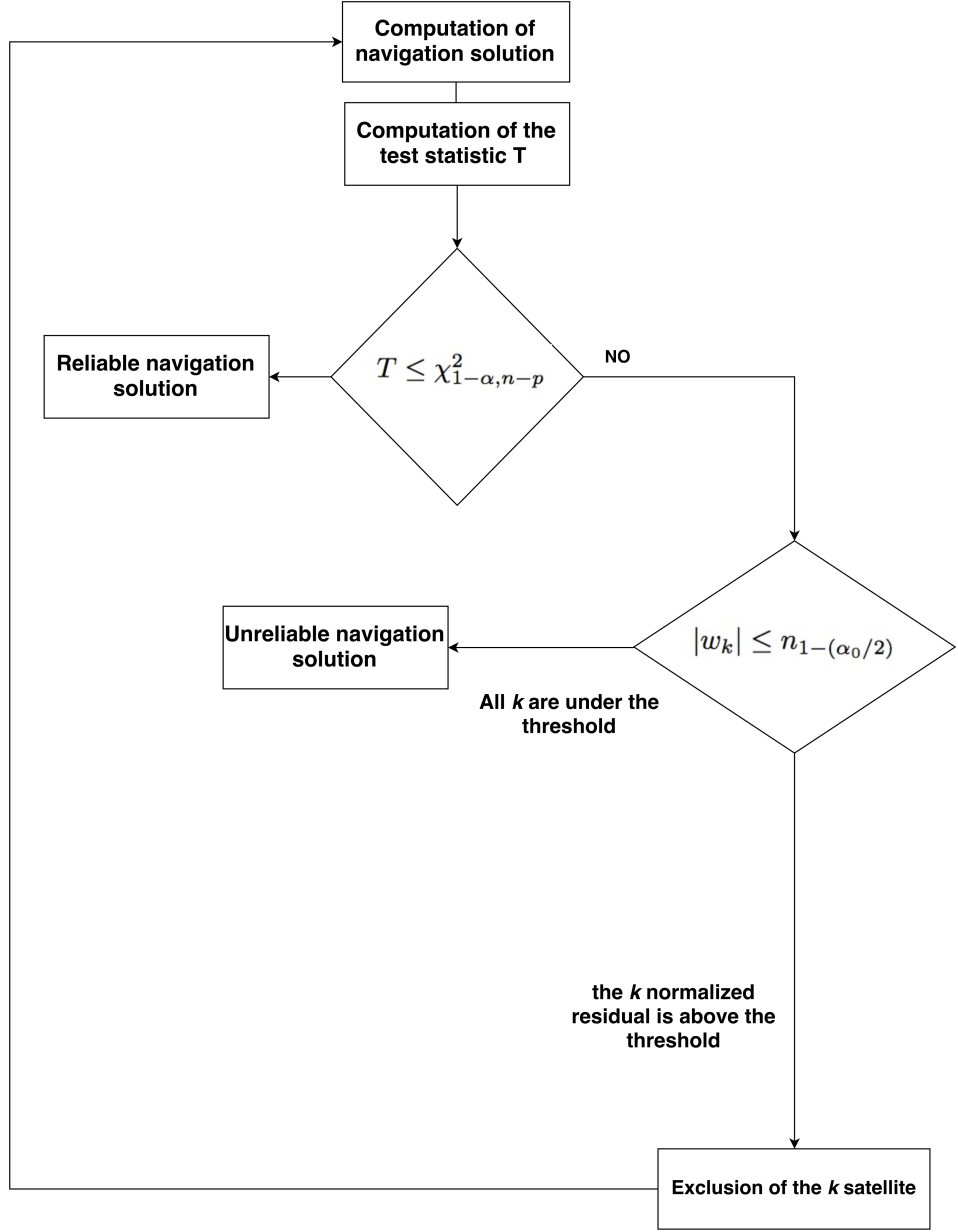
Block diagram of the FDE algorithm.

For the Weighted Least Squares (WLS) solution, if the PR errors are normally distributed with covariance matrix **Σ**, then the residuals are distributed as $\hat{r}∼N\left(0,R\Sigma R^{T}\right)$ [27]. The first test performed is the global test, which checks if a failure exists by calculating the value for the test statistic $T={\hat{r}}^{T}\Sigma^{-1}\hat{r}$. If the error is a Gaussian variable with zero-mean, *T* follows a Chi-square distribution with n−p degree of freedom (DOF), where *n* is the number of measurements and *p* the number of parameters to be estimated. In other words, n−p is the number of redundant measurements. Therefore, once we get *T*, we need to compare it against a threshold value. To do this, we have to establish a certain level of false alarm probability (*α*) and missed detection (*β*). If the current calculated *T* fails the test, the local test is performed to identify which is the faulty satellite. The elements of the residual vector $\hat{r}$ are normalised by the diagonal elements of the covariance matrix of the residual and finally, we get $\hat{w}∼N\left(0,1\right)$. The fault exclusion is performed with the null and alternative hypotheses test with $H_{0}$: $\left|w_{k}\right|\leq n_{1-(\alpha_{0}/2)}$, where $\alpha_{0}$ is the false alarm probability of the local test. The values of the parameters *α*, *β* and $\alpha_{0}$ are linked together and, once two of them are fixed the third is obtained. The *β* parameter is involved for both global and local test (through the non-centrality parameter [28]). The complete sequence of the tests is shown in Figure 10.

### 7.2. Covariance Matrix Uncertainty

In the simulations, the model for the PR error variance is $\sigma_{k}^{2}=a+b\times 10^{-0.1(C/N_{0})}$, where *k* is the satellite index. The model is discussed in [29] and used in many other works [27], where the values *a* and *b* are constant and take into account the degradation caused by the environment. In [28] the authors suggest a=10 m^2^ and b=22,500 m^2^ Hz for lightly degraded signal conditions. The implicit assumptions is that the covariance matrix Σ is diagonal with entries $\sigma_{1}^{2}\cdots \sigma_{k}^{2}$, but this in general is not true and the measurements are correlated, then the matrix entries out of the diagonal are different from zero [30]. In a non-aviation context, like road or railway, the covariance matrix is not generally diagonal [31,32]. In [33] there is an example on how to deal with the uncertain covariance. In this paper we used a diagonal matrix, since the correct definition of a non diagonal matrix is out of the scope of this work.

## 8. SQI and RAIM Interaction

The SQI introduced in the previous section has to be integrated within the algorithms of PVT and RAIM in order to improve the accuracy of the estimated position. The simplest integration method could be to set a threshold SQI_thres_, and to exclude the satellites with SQI < SQI_thres_ from the PVT computation. However, in the case of a low number of satellites in view, the problem of the satellite-user geometry has an impact on the accuracy, and this should be considered in the mechanism of SQI/RAIM/PVT integration. This means that a trade-off has to be found, considering also the effect of the Geometric Dilution Of Precision (GDOP) (or simply Dilution Of Precision (DOP)) [17] , on the estimated position. In fact in some cases the error introduced by the worst DOP (due to a satellite exclusion) could be bigger than the error due to the effect of MPs in some of the satellites in view included in the PVT computation.

In the case of a bad geometry and of strong distortions, instead of removing degraded satellite signals from the PVT, another possible approach is to penalise satellites with a poor signal quality in the WLS solution of the navigation equations, by changing the diagonal noise covariance matrix Σ, as

$$\Sigma_{k,k}\left(t\right)=\sigma_{k}^{2}\left(t\right)+\sigma_{k}^{2}\left(t\right)\gamma \left(1-SQI\left(t\right)\right)$$

where *γ* is a penalty weight. The diagonal entry *k* can assume values from $\sigma_{k}^{2}\left(t\right)$, when SQI = 1 (corresponding to a signal in good conditions), to $\left(\gamma +1\right)\sigma_{k}^{2}\left(t\right)$ when SQI = 0 (corresponding to a strong distortion). In this way, we can reduce the effects of the errors in the PRs projected in the position domain. This method is denoted as P-WLS in the following (where P stands for penalty).

### 8.1. Simulation Results without Using GDOP Data

The purpose of the Section 3 simulations is to show the effects of some MP distortions in the GNSS receiver. In order to validate this method we chose scenarios in which the MP phenomenon had longer durations than those ones from Section 3. We show simulation results obtained by processing these new scenarios, with different numbers of satellites in view, and with MP events that affect specific satellites in the time windows indicated in Table 2. The purpose is to analyze the effects of satellite exclusion and P-WLS, without considering GDOP data. For the PVT computation, the WLS solution is used. The RAIM algorithm used in simulation has a false alarm probability $\alpha =5\times 10^{-5}$ and missed detection probability $\beta =5\times 10^{-5}$. The two simulation scenarios represent the same static position and they have different durations and are presented in Table 2:

- Scenario 1 of duration of 50 s
- Scenario 2 of duration of 90 s

Both scenarios globally have 10 satellites in view.

**Figure 11:**
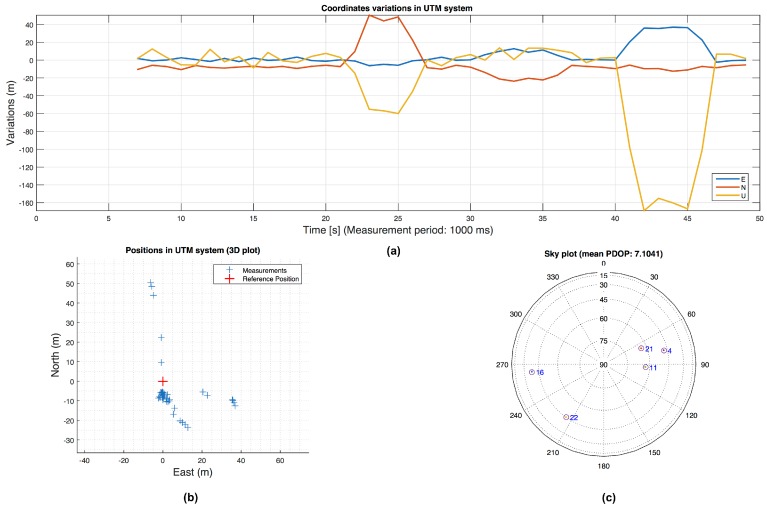
WLS solution of the scenario 1. In (**a**) the position error is shown. The East-North coordinates (**b**) and the skyplot (**c**).

**Figure 12:**
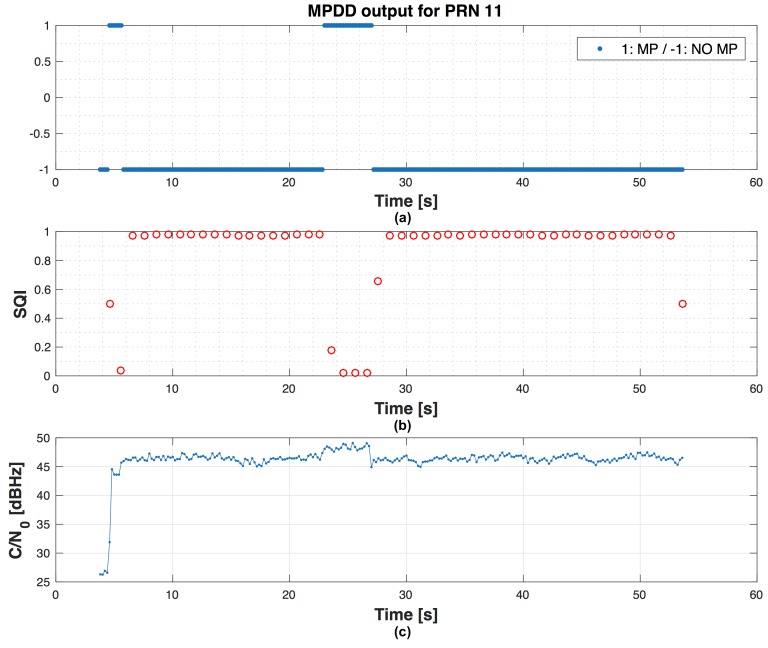
Example related to PRN 11 in scenario 1: the MPDD output (**a**); the SQI (**b**) and the $C/N_{0}$ trend (**c**).

When in the scenarios we consider only 5 chosen satellites in view, obviously FDE cannot be used for exclusion, but the anomalies on PRs can be detected. An example of the position errors in East-North-Up (ENU) coordinates in scenario 1 with 5 satellites, without using RAIM and signal processing assistance, is shown in Figure 11. In Figure 12 the output of the detector, the SQI and the $C/N_{0}$ trend related to PRN 11 in scenario 1 is shown. The Table 3 and Table 4 show the position errors in East-North-Up (ENU) coordinates in scenario 1 and 2 with several values of the penalty weight in the P-WLS method. The performance is also evaluated in terms of MSE over the whole duration of the scenarios. It is possible to observe that especially in scenario 2, where the environment is more degraded than in the scenario 1, the penalty on pseudorange variances can have a strong impact in terms of accuracy expressed in MSE. In scenario 1 the MP affects several PRN in different time windows but with shorter duration, so the penalty weight is between 0 and 1 except for the case with 5 satellites. In fact, in both scenarios another parameter that influences the choice of the penalty weight is the satellites geometry. If most of the satellite signals that we have are not affected by any impairment and also we have a good geometry, penalising the variances may be useless. Finally, the accuracy takes benefit from the penalty weight in a harsh environments.

The results summarised in Table 5 and Table 6 show the effects of the exclusion performed by applying a threshold on SQI. The results have been obtained with SQI_thres_ = 0.7. As it can be seen in the first row of Table 5 relative to the case of 5 satellites, if we exclude one satellite with low SQI, we could get a worse solution due to the bad geometry. This is true with a low number of satellites, but the exclusion leads to an improved solution with a high number of satellites. In summary these results prove that the effect of GDOP has to be considered in the set up of the exclusion algorithms, when the number of satellites in view is very low.

Note that the focus of this paper is to show that fault detection via signal processing techniques is possible and useful especially for land applications where the environment has important effects on the degradation of the signals. For this reason methods for a proper selection of the threshold have not been considered, and will the objective of a future study activity.

### 8.2. GDOP Control

The simulation results just presented motivate the adoption of an exclusion method able to implement a trade-off between the weights of errors removed by signal quality index and weights of the potential errors introduced by the geometry.

A possible approach is to calibrate the dictionary on specific error cases, in order to detect and remove satellites affected by these specific problems. A second approach is to consider that, in a future with a high number of satellites, the risk to degrade the DOP will be lower, but in any case, an additional block of DOP control is convenient for these kinds of algorithms.

We propose here a control, based on a GDOP budget, which tries to remove satellites starting from the ones with lowest SQI and SQI < SQI_thres_ in order to eliminate the distorted signals which could not be detected by the RAIM. Then a GDOP test can be performed, for example by observing if the GDOP after removal exceeds *k* times the GDOP with all the tracked channels. The idea is to combine several effects: calibration of the dictionary depending on the considered environment, and choice of the parameter *k*. In this heuristic way, we can have a kind of control even on the geometry effects, avoiding to introduce wide errors due to the weak geometry. For example, we set *k* = 0.1, so it means that the new GDOP after a possible exclusion cannot exceed 10% of the previous GDOP. By taking in consideration the situation in Table 6 with 6 or 7 satellites, with GDOP control we do not perform an erroneous exclusion and we will get in this way the same results as in not assisted RAIM case (as reported in Table 7). Conversely, with 10 satellites, GDOP control simply permits the additional exclusion performed by SQI.

Another possible approach to avoid problems in case of a poor geometry is to leave the decision on the exclusion to RAIM algorithms but using SQI to identify unreliable navigation solutions. It is possible to declare a navigation solution unreliable when the global test fails but the local test is passed for all the normalised PR residuals (see Figure 10). It means that a faulty situation is detected, but it is impossible to identify which is the faulty satellite and consequently RAIM cannot perform the exclusion. In this situation the RAIM algorithm is not able to provide information related to Vertical or Horizontal Protection Level (VPL and HPL).

**Figure 13:**
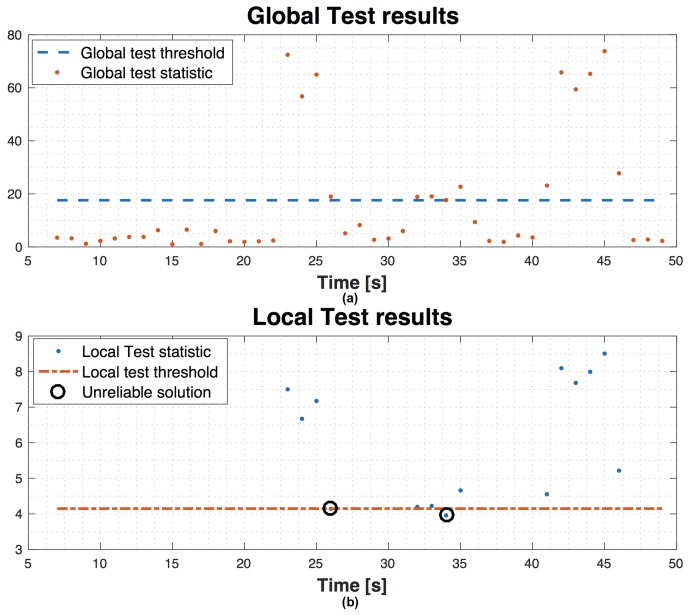
Example of global (**a**) and local (**b**) test on scenario 1 with two unreliable solutions.

**Figure 14:**
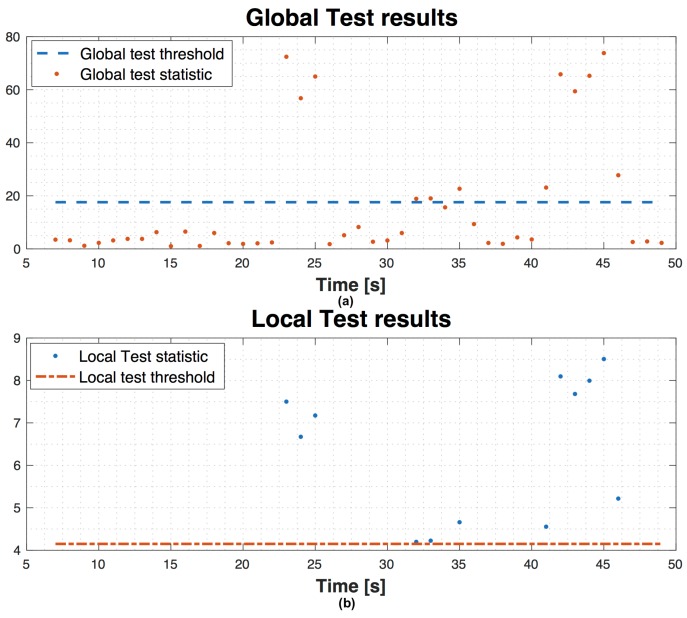
Example of global (**a**) and local (**b**) test assisted by SQI information on scenario 1 without unreliable solutions.

Figure 13 shows test results regarding scenario 1 with 9 satellites in view, without using SQI, with a false alarm probability $\alpha =3.33\times 10^{-5}$ and missed detection probability $\beta =1\times 10^{-2}$. The graph on top is the evolution in time of the global test statistic compared to the global threshold. Then, the local test is performed on the points above the global threshold. In the graph on bottom, the evolution in time of the local test statistic shows that there are only two points under the local threshold (circle points). These values under the local threshold are the navigation solutions which fail the global test, but pass the local one. By using SQI, we want to identify the possible biased PR and exclude it to make reliable the navigation solution. To do this, after the global test failure and when local test is valid, instead of declaring an unreliable solution, we can check the SQI values for each satellite and decide to exclude the satellite with the lowest SQI and repeat again the global and local test (if needed). In Figure 14 the results of the assistance are shown. We observe that there are not points under the threshold in the local test graph, since after the exclusion, the global test is repeated and no more biases are detected and so the local test is not repeated.

## 9. Conclusions

This paper presents a technique to detect the presence of distortions at the tracking stage level of a GNSS receiver. In particular the method has been tailored to MP detection, but can be applied also to other impairments. The specific goal is to assist the modules of integrity monitoring of RAIM. In the near future, the presence of a multi-constellation of satellites like GPS, Galileo, GLONASS, Beidou and of receivers able to demodulate all these signals, will provide an increasing number of satellites in view. This will foster the development of signal processing techniques of FDE, since they perform better with a high number of satellites. In general, this kind of exclusion approach is sensitive to the DOP variation, and the effects of the satellite geometry could be greater than a simple MP phenomenon in terms of accuracy. For this reason a method to combine GDOP control and FDE is also proposed in this paper.

Another way to integrate SQI into RAIM suggested in this work is the exclusion of satellites with lowest SQI in case when RAIM declares a navigation solution as unreliable.

In this work, we have not taken into consideration a possible use of a carrier-smoothing techniques, that might be useful to protect against sporadic MP distortions, because usually they are used in the case of good visibility of the satellites in view.

The proposed technique could be also employed for other kinds of distortion, like spoofing attacks that can bypass RAIM tests. Work is in progress on some modifications of the algorithm, for application of spoofing detection.

## Figures and Tables

**Table 1:** Time windows when MP is present.

Multipath Instants	Duration (s)
10.900–11.300	0.400
27.900–28.100	0.200
42.350–42.650	0.300
50.750–50.900	0.150
53.475–53.525	0.050

**Table 2:** Time windows when MP is present for the scenario 1 and 2.

Scenario	MP Windows (s)	PRN
1	8–16	27
	21–25	11, 22
	30–35	16
	40–45	1, 4, 21, 27
2	20–80	1, 4, 21, 27

**Table 3:** Results for the scenario 1 with increasing penalty weight and different number of satellites.

Number of Satellites	*γ*	E (m)	N (m)	U (m)	MSE (m)
5	0	17.5363	8.36942	37.5979	24.0336
	1	17.5299	8.36801	37.4598	24.0374
	5	17.5148	8.39186	37.1987	23.987
	10	17.5049	8.42931	37.0731	23.9496
8	0	11.6558	9.08143	31.5525	22.7982
	1	11.8193	9.07103	32.3279	22.8998
	5	12.1992	9.09621	34.1857	23.3293
	10	12.4388	9.15166	35.3221	23.6366
10	0	5.82226	8.88567	12.4009	12.7028
	1	5.78051	8.91175	12.2429	12.4587
	5	5.83003	9.15222	12.3956	12.4954
	10	5.94344	9.3618	12.8177	12.7211

**Table 4:** Results for the scenario 2 with increasing penalty weight and different number of satellites.

Number of Satellites	*γ*	E (m)	N (m)	U (m)	MSE (m)
5	0	3.04783	9.27226	57.8219	51.5876
	1	1.77521	6.44012	45.965	41.311
	5	1.98589	11.7037	31.2759	30.1326
	10	2.51859	14.378	26.4405	27.2389
8	0	8.28045	10.9965	41.898	38.7889
	1	4.54109	14.0635	36.4797	34.4765
	5	2.30562	16.053	31.0838	30.0961
	10	3.10298	15.8458	29.9185	28.7643
10	0	7.028	13.9999	37.057	35.5515
	1	3.60098	16.0674	29.853	30.284
	5	1.77159	15.8387	20.8092	23.409
	10	2.20445	14.2902	17.4283	20.2332

**Table 5:** Results for the scenario 1 with *SQI_thres_* = 0.7 and no penalty weight.

Number of Satellites	E (m)	N (m)	U (m)	MSE (m)	PRN Tracked	RAIM	SQI Exclusion
5	12.6207	16.6765	56.8226	36.0848	16, 21, 22, 4, 11	NO	NO
	15.1164	16.3122	67.2206	43.4145		NO	YES
6	6.32411	15.6211	20.869	20.8185	6, 16, 21, 22, 4, 11	NO	NO
	6.019	32.2079	17.1807	21.8183		YES	NO
	6.14697	32.2475	16.1726	21.4553		YES	YES
7	6.47547	14.783	13.7957	17.4422	6, 16, 21, 22, 4, 18, 11	NO	NO
	4.52599	14.696	14.0377	15.9173		YES	NO
	4.62159	14.6788	13.575	15.9742		YES	YES
10	5.82226	8.88567	12.4009	12.7028	6, 16, 21, 22, 4, 18, 11, 1, 19, 27	NO	NO
	3.06137	8.44777	8.70849	10.4545		YES	NO
	3.05162	8.10319	8.40837	10.4058		YES	YES

**Table 6:** Results for the scenario 2 with *SQI_thres_* = 0.7 and no penalty weight.

Number of Satellites	E (m)	N (m)	U (m)	MSE (m)	PRN Tracked	RAIM	SQI Exclusion
5	44.6156	99.3248	170.2897	170.1806	1 , 4 , 21, 27, 19	NO	NO
	159.4347	136.0268	395.6745	323.4530		NO	YES
6	44.6886	90.4397	146.9450	150.3777	1 , 4 , 21, 27, 19, 6	NO	NO
	30.9765	65.6038	109.0453	110.9221		YES	NO
	213.7446	444.4343	242.8570	379.8618		YES	YES
7	1.51801	14.7719	8.40841	15.7991	6, 19, 27, 16, 18, 4, 11	NO	NO
	1.51801	14.7719	8.40841	15.7991		YES	NO
	4.04489	24.2859	12.5928	21.7989		YES	YES
10	6.9509	13.8449	36.6576	34.9215	6, 19, 27, 16, 18, 4, 11, 22, 21, 1	NO	NO
	2.4263	6.2989	26.6406	24.4607		YES	NO
	2.6643	10.0206	8.5329	11.8656		YES	YES

**Table 7:** Results for the scenario 2 with *SQI_thres_* = 0.7, no penalty weight and GDOP control enabled with *k* = 0.1 in case of 6 and 7 satellites in view.

Number of Satellites	E (m)	N (m)	U (m)	MSE (m)	PRN Tracked	RAIM	SQI Exclusion
6	44.6886	90.4397	146.9450	150.3777	1 , 4 , 21, 27, 19, 6	NO	NO
	30.9765	65.6038	109.0453	110.9221		YES	NO
	30.9765	65.6038	109.0453	110.9221		YES	YES
7	1.51801	14.7719	8.40841	15.7991	6, 19, 27, 16, 18, 4, 11	NO	NO
	1.51801	14.7719	8.40841	15.7991		YES	NO
	1.51801	14.7719	8.40841	15.7991		YES	YES
